# SLEEP CHARACTERISTIC OF AN OLDER END OF LIFE (EOL) CAREGIVER: A SINGLE CASE STUDY

**DOI:** 10.1093/geroni/igac059.3079

**Published:** 2022-12-20

**Authors:** Hyeyoung Park, Sunghoon Lee, Amelia Bailey, Yunda Liu, Cynthia Jacelon

**Affiliations:** University of Massachusetts Amherst, Amherst, Massachusetts, United States; University of Massachusetts Amherst, Amherst, Massachusetts, United States; University of Massachusetts Amherst, Amherst, Massachusetts, United States; University of Massachusetts Amherst, Amherst, Massachusetts, United States; University of Massachusetts Amherst College of Nursing, Amherst, Massachusetts, United States

## Abstract

**Background:**

Extensive caregiving work, psychological distress, and caregiving burdens put end of life (EOL) caregivers at great risk of sleep disturbances.

**Methods:**

A single case mixed method design was used to explore older EOL caregivers’ sleep characteristics. Multi-dimensional data was collected through a series of methods including questionnaires (caregiver and care recipient demographics, care recipient symptom severity, caregiver caregiving strain, depressive symptoms, and sleep quality), 14 days of National Sleep Foundation (NSF) sleep diary, 28 days of daily sleep and activity measured by wearable personal sleep monitoring devices (Garmin vivofit 4), and a semi-structured interview. Data was analyzed using thematic content analysis. Findings: A 60 year old female taking care of her 85 years old mother with dementia, kidney cancer, and several other illnesses was recruited for this case study. She showed overall poor sleep quality measured by the Pittsburgh Sleep Quality Index (PSQI, total score: 8), and she slept 6.73 (±1.27) hours per day on average with 52.96% of deep sleep, 41.63% of light sleep, and 5.41% awake times. The caregiver reported that her daily sleep quality depended on her care recipient’s health condition each night. She also described others supporting for her caregiving work as a “double-edged sword,” because she needed to trade some privacy for the additional help.

**Conclusion:**

Further study is needed for EOL caregivers’ poor sleep quality considering external factors and sleep intervention for EOL caregivers should be developed considering these external caregiving contexts.

